# Modulation of anterior cingulate cortex reward and penalty signalling in medication-naive young-adult subjects with depressive symptoms following acute dose lurasidone

**DOI:** 10.1017/S0033291718003306

**Published:** 2019-01-04

**Authors:** Selina A. Wolke, Mitul A. Mehta, Owen O'Daly, Fernando Zelaya, Nada Zahreddine, Hanna Keren, Georgia O'Callaghan, Allan H. Young, Ellen Leibenluft, Daniel S. Pine, Argyris Stringaris

**Affiliations:** 1 Department of Child and Adolescent Psychiatry, Institute of Psychiatry, Psychology, and Neuroscience, King's College London, London, UK; 2 Mood Brain and Development Unit, Emotion and Development Branch, National Institute of Mental Health, National Institutes of Health, MD, USA; 3 Department of Neuroimaging, Institute of Psychiatry, Psychology, and Neuroscience, King's College London, London, UK; 4 Department of Psychiatry, Saint-Joseph University, Beirut, Lebanon; 5 Department of Psychological Medicine, Institute of Psychiatry, Psychology, and Neuroscience, King's College London, London, UK; 6 Section on Mood Dysregulation and Neuroscience, Emotion and Development Branch, National Institute of Mental Health, National Institutes of Health, MD, USA; 7 Section on Development and Affective Neuroscience, Emotion and Development Branch, National Institute of Mental Health, MD, USA

**Keywords:** Anterior cingulate cortex, depression, fMRI, lurasidone, penalty, reward

## Abstract

**Background:**

Aberrations in reward and penalty processing are implicated in depression and putatively reflect altered dopamine signalling. This study exploits the advantages of a placebo-controlled design to examine how a novel D_2_ antagonist with adjunctive antidepressant properties modifies activity in the brain's reward network in depression.

**Methods:**

We recruited 43 medication-naïve subjects across the range of depression severity (Beck's Depression Inventory-II score range: 0–43), including healthy volunteers, as well as people meeting full-criteria for major depressive disorder. In a double-blind placebo-controlled cross-over design, all subjects received either placebo or lurasidone (20 mg) across two visits separated by 1 week. Functional magnetic resonance imaging with the Monetary Incentive Delay (MID) task assessed reward functions via neural responses during anticipation and receipt of gains and losses. Arterial spin labelling measured cerebral blood flow (CBF) at rest.

**Results:**

Lurasidone altered fronto-striatal activity during anticipation and outcome phases of the MID task. A significant three-way Medication-by-Depression severity-by-Outcome interaction emerged in the anterior cingulate cortex (ACC) after correction for multiple comparisons. Follow-up analyses revealed significantly higher ACC activation to losses in high- *v.* low depression participants in the placebo condition, with a normalisation by lurasidone. This effect could not be accounted for by shifts in resting CBF.

**Conclusions:**

Lurasidone acutely normalises reward processing signals in individuals with depressive symptoms. Lurasidone's antidepressant effects may arise from reducing responses to penalty outcomes in individuals with depressive symptoms.

## Introduction

Multiple studies implicate reward and dopaminergic system dysfunction in the pathogenesis of major depressive disorder (MDD). Yet, only few studies use experimentally controlled designs to probe the role of these systems in MDD. Here, we examine the acute effects of lurasidone, a novel D_2_ antagonist with adjunctive antidepressant properties, on neural responding to reward in depression using two functional imaging modalities.

Depressed patients display alterations across several key phases of reward processing. Blunting of neural responses when anticipating or obtaining rewards (Knutson *et al*., 2008; Pizzagalli *et al*., 2009; Keren *et al*., 2018) is associated with anhedonia, while increased reactivity to losses may underlie the behavioural avoidance that is characteristic of depression (Stringaris *et al*., 2015; Luking *et al*., 2016; Engelmann *et al*., 2017; Hevey *et al*., 2017). Recently, a direct link has been found between reduced mid-brain dopamine transporter density and neural activity during reward processing within the mesolimbic pathway in healthy and depressed human participants (Dubol *et al*., 2018).

These findings make reward processing an attractive treatment target. Dopaminergic compounds provide a promising way to manipulate fronto-striatal reward pathways (Pessiglione *et al*., 2006; Jocham *et al*., 2011, 2014; Chowdhury *et al*., 2013; Dean *et al*., 2016; Harmer *et al*., 2017). Surprisingly, however, very few studies have used dopaminergic drugs to probe the association between neural reward signalling and depression. Recently, Admon *et al*. (2017) showed that a single-dose of the dopamine receptor antagonist amisulpride normalised reward processing by increasing reward-related striatal activation and corticostriatal connectivity in depressed individuals. This effect is thought to result from transient increases in dopamine signalling at low amisulpride doses (Schoemaker *et al*., 1997; Admon *et al*., 2017). Strengthening of striatal functioning through dopamine antagonists has been shown before in healthy volunteers (Mehta *et al*., 2003; Handley *et al*., 2013) and is presumed to occur through presynaptic D_2_/D_3_ autoreceptor blockade (Fernandez-Seara *et al*., 2011, Goozee *et al*., 2014).

It may seem counterintuitive that some antipsychotics are antidepressant in augmentation treatment for bipolar and MDD, given that D_2_ antagonism (a central feature of all antipsychotics) is known to suppress reward-related striatal activation, for example, with haloperidol (Pessiglione *et al*., 2006; Pleger *et al*., 2009; Oei *et al*., 2012). However, olanzapine, quetiapine and lurasidone, which are efficacious adjunctive antidepressants [olanzapine (Tohen *et al*., 2003, 2014), quetiapine (Suppes *et al*., 2014; Suttajit *et al*., 2014), lurasidone (Loebel *et al*., 2014*a*, 2014*b*; Nelson *et al*., 2015; Suppes *et al*., 2016*a*, 2016*b*)] differ from haloperidol in their broader profile, including greater serotonergic action. Indeed, blockade of serotonergic 5-HT receptors (5-HT_1A_, 5-HT_2A_, 5-HT_7_) stimulates striatal dopamine release and in addition to this, serotonergic neurons directly impact upon reward (and predominantly aversive) processing (Boureau and Dayan, 2011; Huang *et al*., 2012; Inaba *et al*., 2013; Liu *et al*., 2014; Cohen *et al*., 2015; Hayashi *et al*., 2015; Li *et al*., 2016). However, there are few studies that have assessed modulation of loss anticipation and feedback with antidepressant drugs. The evidence thus far points to a pattern of blunting of aversive events with acute administration of selective serotonin reuptake inhibitors (SSRIs) (McCabe *et al*., 2010; Macoveanu *et al*., 2013, 2014; Macoveanu, 2014), but crucially also with D_2_ antagonists that have anti-depressant properties [amisulpride (Admon *et al*., 2017) and aripiprazole (Bolstad *et al*., 2015)]. These findings raise the intriguing possibility that dopamine antagonists with adjunctive antidepressant properties may exert their effects via *reward* and/or *penalty* signal normalisation.

In this paper, we test whether an acute dose of 20 mg lurasidone, a D_2_ receptor antagonist (Loebel and Citrome, 2015) with demonstrated antidepressant properties in monotherapy and in combination treatment (Loebel *et al*., 2014*a*, 2014*b*; Suppes *et al*., 2016*a*; Goldberg *et al*., 2017), influences reward and penalty signal in depression. Lurasidone was selected because it is the most recently licensed dopamine antagonist with antidepressant properties and there is no information with regards to its effects on brain reward and penalty signalling (Loebel *et al*., 2014*a*, 2014*b*; Nelson *et al*., 2015; Nierenberg *et al*., 2015; Suppes *et al*., 2016*a*, 2016*b*; Goldberg *et al*., 2017). We employ a randomised, placebo-controlled cross-over design with functional magnetic resonance imaging (fMRI) and arterial spin labelling (ASL) imaging acquired on two separate occasions per individual. This design overcomes the limitations of correlational studies through randomisation and experimental manipulation. Since symptoms of MDD fall on a continuous dimension (Angst *et al*., 2000; Ayuso-Mateos *et al*., 2010), we recruited medication-naïve subjects across the range of depression severity, including healthy volunteers, as well as people meeting full-criteria for MDD. This research approach is in line with the Research Domain Criteria framework (Morris and Cuthbert, 2012) [e.g. as in Stringaris *et al*. (2015) where symptom levels are related to the brain measurements]. It also does justice to findings concerning the genetic underpinnings of common mental illness (Plomin *et al*., 2009) as well as current approaches to understanding neural system perturbation in a dimensional way (Matthews and Hampshire, 2016).

Depression is characterised by hyporeactivity to reward (Knutson *et al*., 2008; Forbes *et al*., 2009; Pizzagalli *et al*., 2009; Gotlib *et al*., 2010; Admon *et al*., 2015; Luking *et al*., 2016; Keren *et al*., 2018) and hyperactivity to aversive stimuli (Gotlib *et al*., 2010; Admon *et al*., 2015; Luking *et al*., 2016; Engelmann *et al*., 2017), and thus an antidepressant effect could be brought about by increasing reward, decreasing salience to negative events, or, both simultaneously. Given the relative paucity of literature on processing of losses (Keren *et al*., 2018), our study is designed to interrogate both anticipation and feedback of rewards and penalties. We hypothesise a normalisation of fronto-striatal reward and/or penalty function following acute-dose administration in depression. More specifically, we anticipate that subjects scoring high on depression will show a baseline difference in fronto-striatal activity which will be reverted by acute-dose lurasidone. We first explore the expectation that the dopamine antagonist lurasidone will show striatal blunting during the anticipation phase, in line with numerous findings with D_2_ antagonist drugs (Pessiglione *et al*., 2006; Pleger *et al*., 2009). Although, we note that a structurally similar drug, amisulpride has shown opposite effects (Admon *et al*., 2017). An intriguing question is whether any blunting in reward processing that occurs with these drugs could have beneficial effects when dealing with loss. This is important given findings from serotonergic drugs that show on the one hand blunting of reward processing and on the other, amelioration of negative feedback (McCabe *et al*., 2010; Macoveanu *et al*., 2013, 2014; Macoveanu, 2014), which could underlie its antidepressant effects. In addition, we seek to address a key concern in pharmacoimaging studies, namely that shifts in global or regional cerebral blood flow (CBF) could underlie changes observed in a blood oxygenated level dependent (BOLD) fMRI signal. We therefore also use ASL, an imaging modality that allows the quantification of CBF at rest, to disentangle global and regional CBF changes from a BOLD fMRI signal.

## Materials and methods

### Participants

Forty-three participants (28 female, 15 male) were recruited using the research volunteer recruitment webpage at King's College London, social media and posters at university counselling services across London.

We recruited young people across a range of depression and anhedonia scores in the community as symptoms of MDD are known to fall on a continuum (Angst *et al*., 2000; Ayuso-Mateos *et al*., 2010), allowing us to assess the role of symptom level in reward processing on and off lurasidone (see text and online Figs S2–S4 in the Supplementary Methods). Inclusion criteria restricted recruitment to right-handed individuals 18–25 years of age with no contraindications to MRI, no serious medical conditions and no lifetime substance dependence. Please refer to the online Supplementary Methods for full details of inclusion and exclusion criteria. Table 1 provides demographic and clinical information for the entire sample (*n* = 43). Online Table S1 in the Supplementary Methods provides demographic and clinical characteristics of recruited participants according to depression severity cut-off scores from the Beck's Depression Inventory-II (BDI-II). Participants received £230 in compensation for attending the assessment appointment and both scanning visits, in addition to their winnings from the fMRI task. All participants provided written informed consent, as approved by the Ethics Subcommittee of Psychiatry, Nursing & Midwifery Research (RESC reference number: PNM/13/14-122).

**Table 1.** Demographic and clinical characteristics of participants in a study investigating the effect of lurasidone on reward and penalty processing

Characteristic	Participants (*N* = 43)	
	Mean	s.d. (range)
Age (years)	21.83	2.05 (18–25)
Beck Depression Inventory-II	13.89	12.83 (0–43)
Snaith–Hamilton Pleasure Scale	12.18	8.49 (0–29)
	N	%
Female	28	65.12
Caucasian	36	83.72
Current subthreshold depression	7	16.28
Current MDD	11	25.58
Lifetime MDD	15	34.88
Lifetime MDD and current subthreshold depression	5	16.28
Lifetime MDD and current MDD	10	23.26
Current comorbid anxiety disorders	10	23.26

### Design and procedure

Depression and anhedonia scores were assessed using the BDI-II (Beck *et al*., 1996) and the Snaith–Hamilton Pleasure Scale (SHAPS) (Snaith *et al*., 1995). On the basis of BDI-II scores, participants who were eligible following this screening procedure were invited to the assessment appointment.

**Fig. 1.**
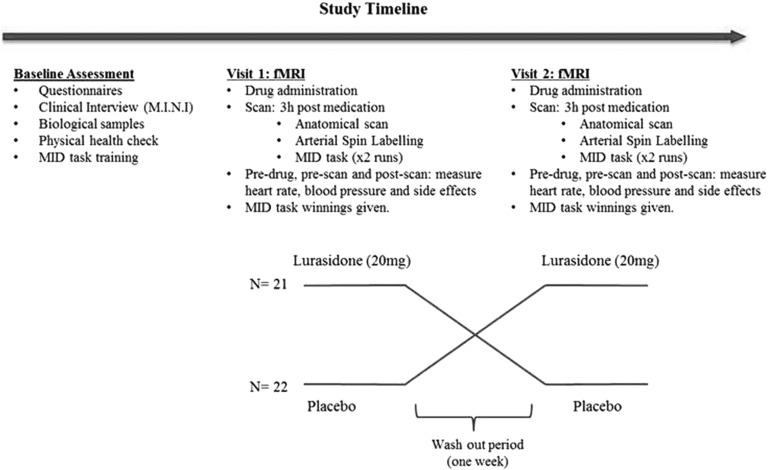
Procedure and timeline for a study investigating the effect of lurasidone on reward and penalty processing.

Figure 1 illustrates the procedure and timeline of the study. At the assessment appointment, participants first completed a pre-MRI safety screening. Participants then completed questionnaires to assess handedness (Edinburgh Handedness Inventory) and IQ (National Adult Reading Test) (Nelson and Willison, 1991). This was followed by the Mini International Neuropsychiatric Interview version 6.0.0 (M.I.N.I.) (Sheehan *et al*., 1998) which assessed past and present mental health disorders. Participants’ height, weight, heart rate, blood pressure and electrocardiogram (ECG) were measured by the experimenter and blood samples (for Full Blood Count and Liver Function Tests) were taken by a study physician. Participants provided a urine sample for drug testing and for pregnancy testing in female participants. Participants were guided through the scanning procedure in a mock scanner and completed training for the Monetary Incentive Delay (MID) task.

If participants fulfilled the inclusion criteria after the assessment appointment, they were invited to take part in two scan days. There was a 97% retention rate in the study and this is illustrated in online Fig. S1 in the Supplementary Methods. Participants were randomised into one of two drug administration orders: placebo-lurasidone (placebo at visit one and lurasidone at visit two), or lurasidone-placebo. Both scan days followed the same schedule. On arrival at the imaging centre, participants had their heart rate and blood pressure measured and filled in two brief questionnaires to measure sedation [Visual Analogue Scale (VAS) (Herbert *et al*., 1976) and state-anxiety (State Trait Anxiety Inventory; STAI) (Spielberger *et al*., 1970)]. Next, the experimenter administered a capsule of either lurasidone (20 mg) or placebo. This dose was selected to minimise post-synaptic D_2_ blockade (la Fougere *et al*., 2005), as in similar studies of related medications (Admon *et al*., 2017). Given the pharmacokinetic profile of lurasidone, the pill was consumed, followed by a 350 calorie meal (Greenberg and Citrome, 2017). Peak plasma levels of lurasidone are reached at approximately 3 h after tablet ingestion and the plasma half-life is 18 h (Greenberg and Citrome, 2017). In order to align the study assessments with peak plasma levels the MRI scan took place 3 h after tablet consumption (Fig. 1). Prior to the MRI scan, 2 h 45 min after drug administration, the experimenter measured participants’ heart rate and blood pressure again, and participants completed the VAS and STAI questionnaires. The scan lasted approximately 1.5 h and included structural scans, ASL and a functional scan acquisition while completing the MID task. After the scan, and approximately 4.5 h after drug administration, the experimenter assessed the participants’ heart rate and blood pressure, the VAS/STAI questionnaires were completed and ECG was collected. Participants were paid in cash for their winnings from the MID task and were discharged.

### fMRI task

The MID task used in the current study was an adaptation of the task from, for example Knutson and colleagues (Knutson *et al*., 2001). The task involves anticipation and receipt of monetary rewards and penalties. The task elicits robust fronto-striatal responses in healthy individuals and has high scan-rescan reliability (Plichta *et al*., 2012; Wu *et al*., 2014). During the anticipation and receipt of monetary reward and penalties, several studies using this task have demonstrated altered fronto-striatal activation in depressed individuals compared with healthy controls (Knutson *et al*., 2008; Pizzagalli *et al*., 2009; Carl *et al*., 2016). This makes the MID task well-suited for the current study and further details are provided in the online Supplementary Methods.

### MRI acquisition parameters

The MRI acquisition parameters are described in the online Supplementary Methods.

### fMRI data analysis

#### ASL pre-processing

Spatial normalisation of the CBF maps was achieved using Automated Software for ASL Processing (ASAP; Mato Abad *et al*., 2016). This pipeline employs the Statistical Parametric Mapping suite (SPM, Functional Imaging Laboratory, University College London, London, UK, version 12 – https://www.fil.ion.ucl.ac.uk/spm). Full details are provided in the online Supplementary Methods.

#### fMRI pre-processing

fMRI data were preprocessed and quality assured using SPM12 in Matlab version (R2016b). This consisted of reorientation to the AC-PC line, slice timing correction, motion correction (Friston *et al*., 1996), multi-channel segmentation and co-registration to each participant's structural image. The normalise estimate & write function within SPM12 was used, with the Montreal Neurological Institute template (MNI152). Smoothing was completed using a Gaussian kernel of 4 mm full-width half-maximum.

#### ASL statistical analysis

To test for statistical significant changes in resting CBF we carried out a paired-sample *t* test, which compared the CBF maps collected after administration of lurasidone against those acquired after placebo. Quantitative measures of global CBF and striatal CBF were extracted for each participant after placebo and lurasidone. The striatal region-of-interest (ROI) was formed by combining anatomically defined binary masks of the caudate, putamen and nucleus accumbens (NAcc) (see online Fig. S7 in the Supplement) (O’Doherty *et al*., 2004). A repeated-measures analysis of covariance (ANCOVA) was performed for global and striatal CBF with the following factors: *Medication* (placebo, lurasidone) as the within-subject variable, *Medication Order* (placebo-lurasidone, lurasidone-placebo) as the between-subject factor and *Depression Severity* (total BDI-II score) as the covariate of interest. To test if changes in baseline CBF were related to the BOLD findings, the change in CBF between the two sessions was entered as covariates in all subsequent analyses. Specifically, the change in CBF values for a given region was used as covariates for the same region in the fMRI analyses.

#### fMRI first-level model

The BOLD signal was modelled with a canonical haemodynamic response function that was convolved with the onset times of task regressors to compute parameter estimates using the general linear model (GLM) at the single-subject level. The GLM included nine task-related regressors: passive condition, three cues (neutral, win, loss) and five outcomes [with (win outcome following win cue), missed win (no-change outcome following a win cue), loss (penalty outcome following a loss cue), avoided loss (no-change outcome following a loss cue) and neutral outcome (no-change outcome following a neutral/no-incentive cue)]. High-pass temporal filtering (128 s cut-off) was used to remove low-frequency artefacts. Estimated movement parameters were added to the design matrix. These included six rigid-body movement parameters, a regressor accounting for frame-wise displacement (i.e. the 3D movement from volume 1–2, 2–3 etc.), and additional binary regressors to indicate image volumes with spikes greater than 1 mm, and images either side of the spike (i.e. motion scrubbing and padding). Movement analyses are described in the online Supplementary Methods.

### fMRI statistical analysis

#### Anticipation and outcome

Following previous findings that depression is associated with differential fronto-striatal abnormalities in response to anticipation *v.* receipt of monetary outcomes (Pizzagalli *et al*., 2009) statistical analyses were separately conducted for the cue and outcome phases of the task.

To test *a priori* hypotheses regarding fronto-striatal responses to the anticipation and outcome of reward and penalty, we conducted a ROI analysis. Mean activations were extracted from seven bilateral anatomical masks of the caudate, putamen, NAcc, orbitofrontal cortex (OFC), anterior cingulate cortex (ACC), insula and amygdala for each participant for the following contrasts of interest: (i) anticipation neutral > baseline, (ii) anticipation win > baseline, (iii) anticipation loss > baseline, (iv) *Reward Outcome*: feedback win > missed win and (v) *Penalty Outcome*: feedback loss > avoided loss. This analytic approach has been used previously (Admon *et al*., 2017) and mitigates possible spillover effects of cue type on the neural responses to outcomes. Masks were collapsed across hemispheres because hemispheric effects on task activation were non-significant and because of the high correlation between hemispheric ROIs. To avoid circular analysis (Kriegeskorte *et al*., 2009), whole regions from atlas toolboxes in SPM12 were used (see online Fig. S7 in the supplementary data). These ROIs were chosen in accordance with meta-analytical findings of the neural correlates of reward and penalty processing (Diekhof *et al*., 2012; Bartra *et al*., 2013; Zhang *et al*., 2013).

For the anticipation phase of the task, a repeated-measures ANCOVA was performed for each ROI with the following factors: *Medication* (placebo, lurasidone) and *Anticipation Cue* (neutral, win, loss) as within-subject variables, *Medication Order* as the between-subject factor, and *Depression Severity* (total BDI-II score) as the covariate of interest.

To test our hypothesis regarding normalisation of reward and/or penalty responses, we conducted a repeated measures ANCOVA for each ROI. This included the factors: *Medication* (placebo, lurasidone) and *Outcome Type* (reward, penalty) as within-subject variables, *Medication Order* as the between-subject factor, and *Depression Severity* (total BDI-II score) as the covariate of interest. We predicted that normalisation responses in depressed individuals on lurasidone would be captured by a *Medication*-by-*Depression Severity*-by-*Outcome Type* interaction. We expected to find no effect of *Medication Order*.

In order to examine further the drug effects on the neural signal, we examined how the difference in neural activity (*Δ*_neural activity_) between placebo and lurasidone in each ROI varied across depression scores. For this, Pearson correlation coefficients were estimated.

To complement our primary dimensional analyses (using a continuous measure of depression), we also examined our hypothesis regarding normalisation of responses using categorical groups in a repeated measures ANOVA model (Fig. 4). We used severity cut-off scores for the BDI-II (Beck *et al*., 1996, Krefetz *et al*., 2002, Kumar *et al*., 2002) to compare individuals with *low depressive symptoms* [total BDI-II score: 0–16 (normal-mild mood disturbance), *n* = 24] to individuals with *high depressive symptoms* [total BDI-II score: 17–43 (borderline-severe depression), *n* = 18] on placebo and lurasidone (Strober *et al*., 1981; Barrera and Garrisonjones, 1988; Whitaker *et al*., 1990; Ambrosini *et al*., 1991; Marton *et al*., 1991; Canals *et al*., 2001).

For all of the above ROI analyses, the threshold for statistical significance was set at (*p* < 0.007) following Bonferroni adjustment for seven multiple ROI comparisons. We also tested the association between dimensional anxiety scores and brain activation in an ANCOVA, with *Anxiety Severity* (total score on the anxiety subscale of the Hospital Anxiety and Depression Scale) as the covariate of interest.

In order to model the effects of lurasidone and depression status beyond the fronto-striatal network targeted in the ROI analyses, exploratory whole brain analyses were also conducted (see the online Supplementary Methods and Results).

## Results

### Behavioural results

A repeated measures ANCOVA with *Medication* (placebo or lurasidone) and *Cue Type* (reward, penalty, neutral) as the within-subject variables, *Medication Order* (placebo-lurasidone, lurasidone-placebo) as the between-subject variable and *Depression Severity* (total BDI-II score) as the covariate of interest was completed for (i) *Total Winnings*, (ii) *Mean Reaction Time* (RT) and (iii) *Accuracy*. Performance data are presented in online Table S2 in the Supplementary Results. In all analyses, there were no effects of *Medication Order* or interactions with *Medication Order* (all *p* values > 0.050). In all analyses there were no significant three-way interactions between either (i) *Total Winnings*, (ii) *Mean RT* or (iii) *Accuracy* and *Medication* and *Depression Severity.* Significant two way interactions between *Cue Type* and *Mean RT*, and *Cue Type* and *Accuracy* are presented in the online Supplementary Results. We also examined the effect of *Medication*, *Medication Order* and *Depression Severity* on the change in *Sedation* ratings (total VAS scores) and *State-anxiety* ratings (total STAI score) from pre-drug administration (Measure 1) to peak-of-drug (Measure 2). There were no significant main effects or interactions (all *p* values > 0.050) (please refer to the online Supplementary Results).

## Reward processing (blood-oxygen-level dependent signal) results

### Response to outcomes

#### Primary analyses

These primary analyses are conducted with depression measured as a continuous variable. In order to test the hypothesis that lurasidone would increase activation to reward outcomes and decrease responses to penalties in depressed individuals, we conducted a repeated-measures ANCOVA. *Medication* (placebo, lurasidone) and *Outcome Type* (*Reward Outcome v. Penalty Outcome*) were the within-subject variables, *Medication Order* was the between-subject factor and *Depression Severity* (total BDI-II score) was the covariate of interest (*n* = 40). Three participants were excluded from the analyses (please refer to the online Supplementary Results). The repeated measures ANCOVA revealed a significant *Medication*-by-*Depression Severity*-by-*Outcome Type* interaction in the ACC (*F* = 8.10, df = 1, 37, *p* = 0.007), after passing Bonferroni adjustment for seven multiple ROI comparisons. The interaction fell short of Bonferroni-adjusted significance in the OFC (*F* = 4.47, df = 1, 37, *p* = 0.041) and insula (*F* = 4.90, df = 1, 37, *p* = 0.033). There were no significant interactions with *Medication Order* (all *p* values >0.050).

To understand the significant three-way interaction, we conducted two repeated-measures ANCOVAs for *Reward Outcome* (*n* = 41) and *Penalty Outcome* separately (*n* = 41 after excluding outliers, please refer to online Supplementary Results).

**Fig. 2.**
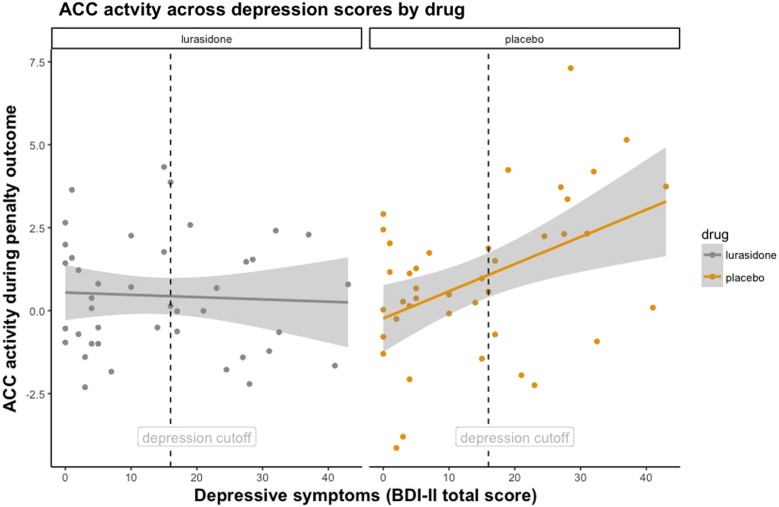
Facet plot illustrating ACC response during Penalty Outcome across continuous depression scores under lurasidone and placebo. Dashed vertical line denotes depression severity cut-off score on the BDI-II.

**Fig. 3.**
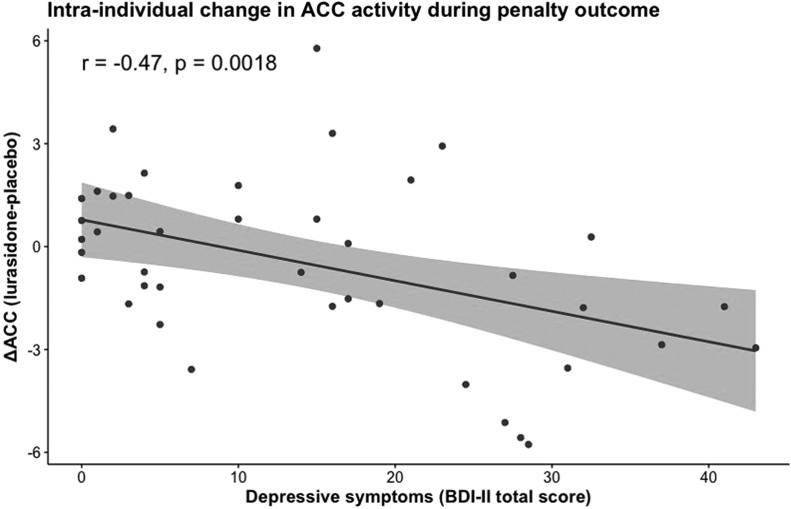
Intra-individual change in penalty related ACC activity (the difference between neural activity under lurasidone and placebo) as a function of continuous depression scores.

This revealed a significant *Medication*-by-*Depression Severity*-by-*Penalty Outcome* interaction in the ACC (*F* = 11.98, df = 1, 38, *p* = 0.001). Figure 2 demonstrates that under placebo, individuals with higher depressive symptoms had greater ACC activity during penalty outcomes. However, this trend was not found under lurasidone. Put simply, brain activity to penalties in the ACC in individuals with elevated depression scores under lurasidone, but not placebo, resembles brain activity of individuals with low depressive symptoms. In keeping with this result, we found that ΔACC (the difference between neural activity under lurasidone and placebo) was negatively correlated with depression severity. Figure 3 illustrates the finding that the absolute difference in neural activity between lurasidone and placebo increased as a function of depression scores.

A similar pattern of results, namely a signal normalisation, was found in the OFC (*F* = 4.94, df = 1, 37, *p* = 0.032), but the interaction fell short of significance after Bonferroni adjustment (see the online Supplementary Results).

We then examined the *Medication*-by-*Depression Severity*-by-*Reward Outcome* interaction across the seven ROIs. This also displayed a pattern of signal normalisation, although in an opposite direction to *Penalty Outcome*, as lurasidone had its strongest effect of *increasing* responses to reward outcomes in individuals with high depression severity. This trend fell short of significance in the NAcc (*F* = 4.87, df = 1, 38, *p* = 0.033) and ACC (*F* = 5.92, df = 1, 37, *p* = 0.020) following Bonferroni correction.

#### Secondary analyses

Complementing the primary (continuous variable) analyses, we sought to replicate our results using categorical analyses. A repeated-measures ANOVA with *Medication* (placebo, lurasidone) and *Outcome Type* (*Reward Outcome v. Penalty Outcome*) as the within-subject variables and *Depression Group* (low *v.* high depressive symptoms) and *Medication Order* as the between-subject factors (*n* = 40), revealed a significant *Medication*-by-*Depression Group*-by-*Outcome Type* interaction in the ACC (*F* = 8.68, df = 1, 38, *p* = 0.005).

**Fig. 4.**
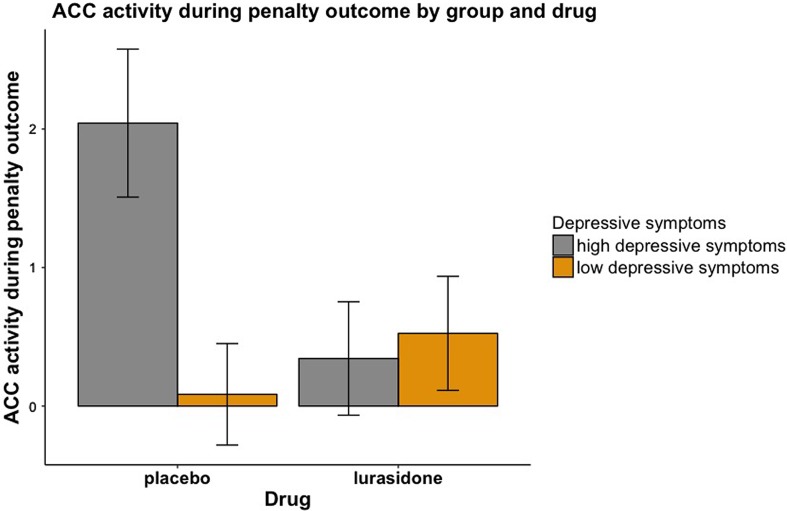
Box plot illustrating ACC Response to Penalty Outcomes (loss > avoided loss). Depression severity cut-off scores from the BDI-II, with individuals with low depressive symptoms (total BDI-II score: 0–16, *n* = 24) *v.* high depressive symptoms (total BDI-II score: 17–43, *n* = 18).

Figure 4 illustrates these findings using BDI-II cut-off scores, with individuals with low depressive symptoms (total BDI-II score: 0–16, *n* = 24) *v.* high depressive symptoms (total BDI-II score: 17–43, *n* = 18). Post-hoc *t* tests showed that participants with high depressive symptoms receiving placebo had significantly greater ACC activation to *Penalty Outcomes* than participants with high depressive symptoms receiving lurasidone (*T* = 2.17, df = 19, *p* = 0.043), and participants with low depressive symptoms receiving placebo (*T* = 2.32, df = 37, *p* = 0.026). There was no significant difference between individuals with high BDI-II scores on lurasidone and individuals with low BDI-II scores on placebo (*T* = 0.48, df = 37, *p* = 0.634). Together, these findings indicate that brain activity to penalties in the ACC in individuals with elevated depression scores under lurasidone, but not placebo, resembles brain activity of healthy volunteers.

To summarise, across reward and penalty outcomes, lurasidone had its strongest effect of increasing responses to reward outcomes and decreasing responses to penalty outcomes in individuals with high depression severity (Figs 2–4). The pattern and significance of the results remained when the outliers were included in the analysis (see the online Supplementary Results).

#### Response to cues

In contrast to the outcome results, there were no significant interactions with depression in the anticipation phase of the task. Instead, the repeated measures ANCOVA revealed a significant *Medication*-by-*Anticipation Cue* interaction in the ACC (*F* = 8.16, df = 2, 72, *p* = 0.001) and caudate (*F* = 7.78, df = 2, 72, *p* = 0.001). Post-hoc tests show that lurasidone reduced responses to win and loss cues *v.* placebo, and increased responses for neutral cues in the ACC and caudate. This fell short of significance in the OFC (*F* = 3.94, df = 2, 72, *p* = 0.024) and amygdala (*F* = 3.85, df = 2, 72, *p* = 0.026).

Anxiety severity analyses and exploratory whole-brain findings for the anticipatory and outcome phases of the task are presented in the online Results Section of the Supplementary data.

#### Cerebral blood flow (CBF)

**Fig. 5.**
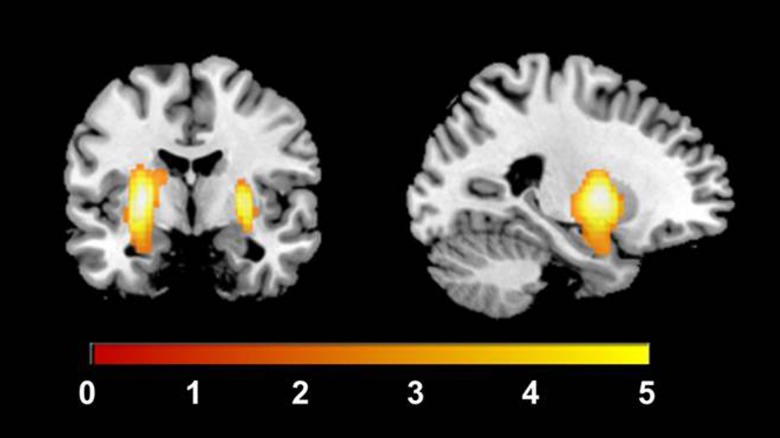
Increased CBF in bilateral putamen for lurasidone relative to placebo during rest in the whole sample (*n* = 43). Significant at the peak level whole-brain analyses, family-wise error-corrected (left putamen *x* = −26, *y* = −4, *z* = 2, *t* = 6.15: *p* = 0.002, right putamen *x* = 28, *y* = −2, *z* = 2, *t* = 5.50: *p* = 0.015). Bar represents *T*-value.

In order to ensure that the BOLD results in the ACC were independent of changes in underlying CBF, we tested the effects of acute lurasidone administration on global and regional blood flow. As shown in Fig. 5, a paired-samples *t* test across the whole-brain showed that lurasidone increased CBF in bilateral putamen relative to placebo during rest in the whole sample (*n* = 43). Significant increases in blood flow were not observed in the ACC. The repeated measures ANCOVA revealed that the extracted global and striatal CBF values were not related to *Depression Severity* (*F* = 0.02, df = 1, 40, *p* = 0.903), *Medication Order* (*F* = 0.44, df = 1, 40, *p* = 0.903), or any three-way interactions with these respective factors (*F* = 0.01, df = 1, 40, *p* = 0.952); (*F* = 1.10, df = 1, 40, *p* = 0.300). The change in CBF values for each of the seven ROIs were extracted and used as covariates for the same region in the fMRI BOLD analyses. This did not lead to any changes in the results: non-significant results remained non-significant and significant results remained significant. In particular, the *Medication*-by-*Depression Severity*-by-*Outcome Type* interaction in the ACC (*F* = 8.13, df = 1, 36, *p* = 0.007).

## Discussion

In this study, we compared the effects of lurasidone and placebo on neural responding to reward and penalties in medication-naïve young-adult subjects across the range of depression severity. During the anticipation phase of the task, we found that lurasidone reduced responses to win and loss cues *v.* placebo, and increased responses for neutral cues in the ACC and caudate across the entire sample (i.e. regardless of depression severity). We found that brain activity in the ACC to *Penalty Outcomes* in individuals with high symptoms of depression under lurasidone, but not placebo, resembled brain activity of individuals with low symptoms of depression. Specifically, lurasidone *reduced* ACC signalling to negative feedback in young people with elevated depressive symptoms. Increased regional and global blood flow under lurasidone did not drive the BOLD findings. These results provide evidence for abnormalities in neural reward-penalty systems in depression and highlight the potential of targeted pharmacological treatments (dopaminergic agents) to normalise penalty related processing in depression.

Our findings are consistent with the notion that acute dose of drugs with antidepressant properties, either as used in monotherapy or combination treatment, can have an effect on brain processes implicated in depression (Harmer *et al*., 2017). For example, SSRIs reduce negative bias and amygdala response to negative emotional stimuli (Harmer *et al*., 2009; Murphy *et al*., 2009). In our study, the effects of normalisation where localised to the ACC, a region that integrates diverse striatal and prefrontal functions (Haber and Knutson, 2010). For example, the ACC and ventral striatum (VS) show functional connectivity at rest (Pan *et al*., 2017) and input from the ACC to the VS allows for flexible deployment and adaptation of behaviour to changing circumstances (Holroyd and Coles, 2002; Walton *et al*., 2007; Alexander and Brown, 2011; Holroyd and Yeung, 2012; Walsh and Anderson, 2012; Holroyd and Umemoto, 2016; Umemoto and Holroyd, 2016; Shahnazian and Holroyd, 2017). Electrophysiological (electroencephalogram) studies have shown that the feedback negativity (FRN), an event-related potential which indicates the early appraisal of feedback and appears larger following the presentation of negative feedback, has its origins in the ACC (Gehring and Willoughby, 2002; Holroyd and Coles, 2002; Holroyd *et al*., 2004; Hajcak *et al*., 2005; Yeung *et al*., 2005). Specifically, an FRN signal may be generated as ACC neurons shift from encoding expected to actual outcomes (i.e. a prediction error signal) (Hyman *et al*., 2017). In our study, participants with higher depression severity on placebo showed greater ACC response to negative feedback. This is congruent with evidence of heightened sensitivity to negative outcomes in depression and its association with elevated loss-related signals in the ACC, and connected regions such as the anterior insula and striatum.

It has been postulated that increased ACC activity in depressed individuals to loss outcomes reflects biased stimuli representations that mediate choice behaviour, including preferential attention, planning and self-referential processing towards losses (Sylvester *et al*., 2003; Grimm *et al*., 2009; Gotlib *et al*., 2010). A normalisation of ACC response in depressed individuals on lurasidone therefore suggests that lurasidone may act to decrease salience and processing of loss events.

Inter-individual differences between low and high depression severity subjects could account for the findings that lurasidone attenuated response to penalty outcomes in individuals with high depression severity only. Indeed, depression is associated with baseline differences in availability and function of 5-HT and/or D_2_ receptors and reductions in binding relative to healthy volunteers (Suhara *et al*., 1992; Yatham *et al*., 1999; Sheline *et al*., 2004; Yatham *et al*., 2005). Thus, in accordance with previous findings that more divergent patterns of reward/penalty processing at baseline are associated with greater post-intervention change (Vrieze *et al*., 2013; Rice *et al*., 2015; Burkhouse *et al*., 2016; Walsh *et al*., 2016), it could be that subjects with more severe depressive symptoms have more ‘room for improvement’ following acute lurasidone administration.

In addition to attenuating penalty outcome responses, lurasidone reduced neural responses in the ACC and caudate during the anticipation of loss and reward cues across the entire sample (i.e. regardless of depression severity). This is in line with studies showing attenuated reward-related striatal activation during reward anticipation and decision making with D_2_ antagonist haloperidol (Pessiglione *et al*., 2006; Pleger *et al*., 2009). However, no effect has been reported for prediction of losses (Pessiglione *et al*., 2006). There are various mechanisms which could account for the finding in this study, all of which are speculative at the moment. First, lurasidone may modulate tonic and phasic dopamine firing either directly by D_2_ antagonism or indirectly via antagonism at serotonergic 5-HT receptors (5-HT_2A_, 5-HT_7_). Antagonism at D_2_ receptors could act to block and reduce dopamine release, thereby also attenuating the BOLD signal. Alternatively, lurasidone may, at low doses, like amisulpride increase striatal dopamine release by preferentially blocking presynaptic dopamine auto-receptors. Increased dopamine availability may act to increase tonic levels of dopamine, in turn decreasing the phasic firing of dopamine neurons and the sensitivity of the dopamine reward system (Grace, 1991), thereby potentially reducing BOLD signal to anticipation cues. Although it must be noted that ascribing the changes seen to one or more receptor systems is highly speculative as the precise mechanism by which BOLD signal is modulated cannot be determined with fMRI alone.

It is notable, that in line with previous studies utilising dopamine antagonists (Lahti *et al*., 2003; Lahti *et al*., 2005; Handley *et al*., 2013; Goozee *et al*., 2014), we show here that lurasidone increased striatal CBF at rest. Increases in blood flow following antipsychotic lurasidone administration may be related to increased neuronal metabolism in striatal areas due to the large density of D_2_ receptors (Goozee *et al*., 2014), with blockade of D_2_ receptors in the striatum potentially resulting in disinhibition of D_2_ receptor-containing medium spiny neurons (Fernandez-Seara *et al*., 2011). Our results showed that the penalty and reward-related findings were unchanged after controlling for baseline shifts in global and striatal CBF, and highlight the utility of multi-modal fMRI in identifying if the effects of the drug administered are indeed neuronal.

Our study also showed a pattern in which lurasidone potentiated striatal (NAcc) activity to reward outcomes in young adults with elevated depressive symptoms. These findings did not survive stringent correction for multiple comparisons and should therefore be interpreted with appropriate caution. We note, that these results are in keeping with recent findings by Admon *et al*. (2017) who showed that a single-dose of the dopamine receptor antagonist amisulpride normalises reward processing by increasing reward-related striatal activation and connectivity between the striatum and mid-cingulate cortex in depressed individuals.

This study has several strengths. First, we tested the association between reward processing and depression using randomisation and experimental manipulation, thereby overcoming several of the limitations of correlational studies in drawing causal inferences. Second, the cross-over, within-subject design affords higher statistical power than a parallel design by minimising subject variance as each individual acts as their own control, and increasing the drug variance. Third, we recruited medication-naïve subjects across the range of depression severity, thus avoiding the confound of medication (Pessiglione *et al*., 2006; Abler *et al*., 2007).

This study also has limitations. Caution should be exercised with the interpretation of our results as ‘normalising’. In the absence of any behavioural effect there is no evidence for better performance of the task on lurasidone, there is no clear main effect of depressive symptoms on the task (i.e. no deficit to improve) and no other triangulating measure of response to negative outcomes which can be linked to function. Nevertheless, it could be argued that lurasidone changes an activity in the ACC that might be beneficial. Further studies need to address both the behavioural deficit and the neural changes in parallel. This has proven challenging as it requires alignment between different levels of explanation including task, neural, clinical and behavioural (Keren *et al*., 2018).

Our study was not designed to capture changes in depressive symptoms following lurasidone and therefore it is unclear how these would correlate with brain responses. However, our strategy of searching for the signal of an intervention in the first place is consistent with current recommendations to boost drug discovery (Krystal and State, 2014). The next piece of information which would be needed to infer causality, is whether lurasidone-induced neural changes (reduced penalty related ACC signalling and increased reward-related NAcc signalling) predict a decline in depressive and anhedonic symptoms (Shiroma *et al*., 2014; Godlewska *et al*., 2016). This would require longer-term lurasidone treatment in longitudinal studies with assessment of pre-post changes in behavioural and neural responses. Antidepressants seem to exacerbate reward deficits early in treatment (Kumar *et al*., 2008; McCabe *et al*., 2010; Marutani *et al*., 2011) prior to normalisation following longer-term (2–6 week) treatment (Stoy *et al*., 2012; Scholl *et al*., 2017; Walsh *et al*., 2017). Thus, in line with longer-term dosing studies, repeated dosing with lurasidone could lead to increasing anticipation of rewards with more chronic exposure to the drug. Although speculative, one could predict a behavioural activation model of the antidepressant mechanism of action of lurasidone, with normalisation of responses to outcomes (consummation), prior to a normalisation of neural anticipatory signals with longer-term treatment (Dimidjian *et al*., 2011).

We note that we used two contrasts for the outcome type: reward and penalty outcome. Whilst this is standard in the literature in similarly designed studies (Admon *et al*., 2017), an alternative modelling could be four levels: reward, missed reward, penalty and avoided penalty outcomes relative to no incentive outcomes. In addition, the recruitment was designed for analysis of depressive symptoms as a continuum, and as such any analysis of those with higher scores contrasted with lower scores may be underpowered. Interestingly we were able to replicate the results of Admon *et al*. (2017), as lurasidone potentiated striatal (NAcc) activity to reward outcomes using such categories but this did not survive correction for multiple comparisons.

In conclusion, our study shows that an acute dose of dopaminergic agent, lurasidone, transiently decreased penalty related ACC activity in individuals with high symptoms of depression. These findings suggest that modulation of dopamine transmission may help to normalise processing of negative outcomes in depressed individuals through the alteration of ACC signalling. Thus, ACC signalling may provide a new target for engagement in future drug development studies. Using an experimental medicine design such as the one used in this study, could help identify relevant compounds which could then be tested further in using longer-term follow-up.

## Supplementary material

For supplementary material accompanying this paper visit https://doi.org/10.1017/S0033291718003306. click here to view supplementary material
