# Supplementary material for: Modulation of anterior cingulate cortex reward and penalty signalling in medication-naive young-adult subjects with depressive symptoms following acute dose lurasidone
Source: Psychol Med. 2019 Jan 4;49(8):1365–77. doi: 10.1017/S0033291718003306 (PMC6518385; doi:10.1017/S0033291718003306)
Supplement: Supplementary file 1 [file S0033291718003306sup001.docx]

**Data Supplement**

**Supplementary Methods**

Participants and procedure: We recruited young people across a range of depression and anhedonia scores in the community as symptoms of MDD are known to fall on a continuum, allowing us to assess the role of symptom level in reward processing on and off lurasidone (see Figures S2-S4). This was completed by prospective candidates first being pre-screened on the BDI and SHAPS. On the basis of BDI-II and SHAPS scores, prospective candidates were selected to participate in a telephone interview to briefly assess relevant psychiatric, medical and neurological history. Participants who were eligible following this screening procedure were invited to the assessment appointment. Exclusion criteria for all participants included: left-handed individuals outside the 18-25 age range, having a current or previously diagnosed psychiatric disorder, except depression or comorbid depression-anxiety disorder, having one or more immediate family members with a history of schizophrenia, autism or bipolar disorder, and, a history of pharmacological treatment for a psychiatric disorder or any such current treatment, contraindications to MRI (e.g., metal implants, pacemakers, claustrophobia etc.), a serious or unstable medical illness (e.g. diabetes, cardiovascular, respiratory, endocrine, neurologic or hematologic disease), history of gastrointestinal, hepatic, or renal disease or other condition known to interfere with absorption, distribution, metabolism or excretion of medications, history of seizures, acute illness two weeks before the start of the study, pregnancy, clinically significant abnormalities in Full Blood Count (FBC) and Liver Function tests (LFT), use of prescribed medication in the 3 weeks prior to enrolment or non-prescription medication (other than 1g paracetamol/24 hours) or herbal preparations in the previous seven days, receipt of another new chemical entity in the four months before dosing, or participation in another study within three months before the start of the present study, (or within one month for a non-invasive methodology study where no drugs were given), blood or needle phobia, lifetime substance dependence, being a cigarette smoker (including e-cigarettes), positive urine drug test (benzoylecgonine, d-amphetamines, d-methamphetamines, THC, Morphine) and having taken illicit drugs (six months), alcohol (24 hours), caffeine (six hours), or nicotine (four hours) before scanning. To avoid craving effects, we recruited participants who were non-smokers and consumed less than three cups of coffee per day.

Diagnostic criteria as assessed by the Mini International Neuropsychiatric Interview version 6.0.0 (M.I.N.I)

Table 1 in the main text provides demographic and clinical information organized by high- (Total BDI-II scores: 17-43 (borderline- severe depression), n=18) and low (Total BDI-II scores: 0-16 (normal-mild mood disturbance), n=24) depression severity groups. Table 1 also demonstrates the convergence between BDI-II scores and depression diagnoses as assessed by the Mini International Neuropsychiatric Interview version 6.0.0 (M.I.N.I) (Sheehan *et al*, 1998).

Individuals were included in a subthreshold depression group if they self-reported having experienced, in the past two weeks, at least three depressive symptoms including at least one core symptom (abnormally depressed, irritable mood, or loss of interest) and two or more other DSM-IV depressive symptoms, without fulfilling criteria for MDD in terms of duration, symptom number, or significant impact on functioning (Lewinsohn *et al*, 2000). MDD was diagnosed if the individual self-reported having experienced at least five depressive symptoms including at least one core symptom (abnormally depressed or loss of interest) most of the day, nearly every day for the past two weeks, with significant functional impairment. Comorbid simple phobia, panic and agoraphobia, social anxiety disorder and generalized anxiety disorder were allowed. MINI interview scores and diagnoses were reviewed by a consultant psychiatrist.


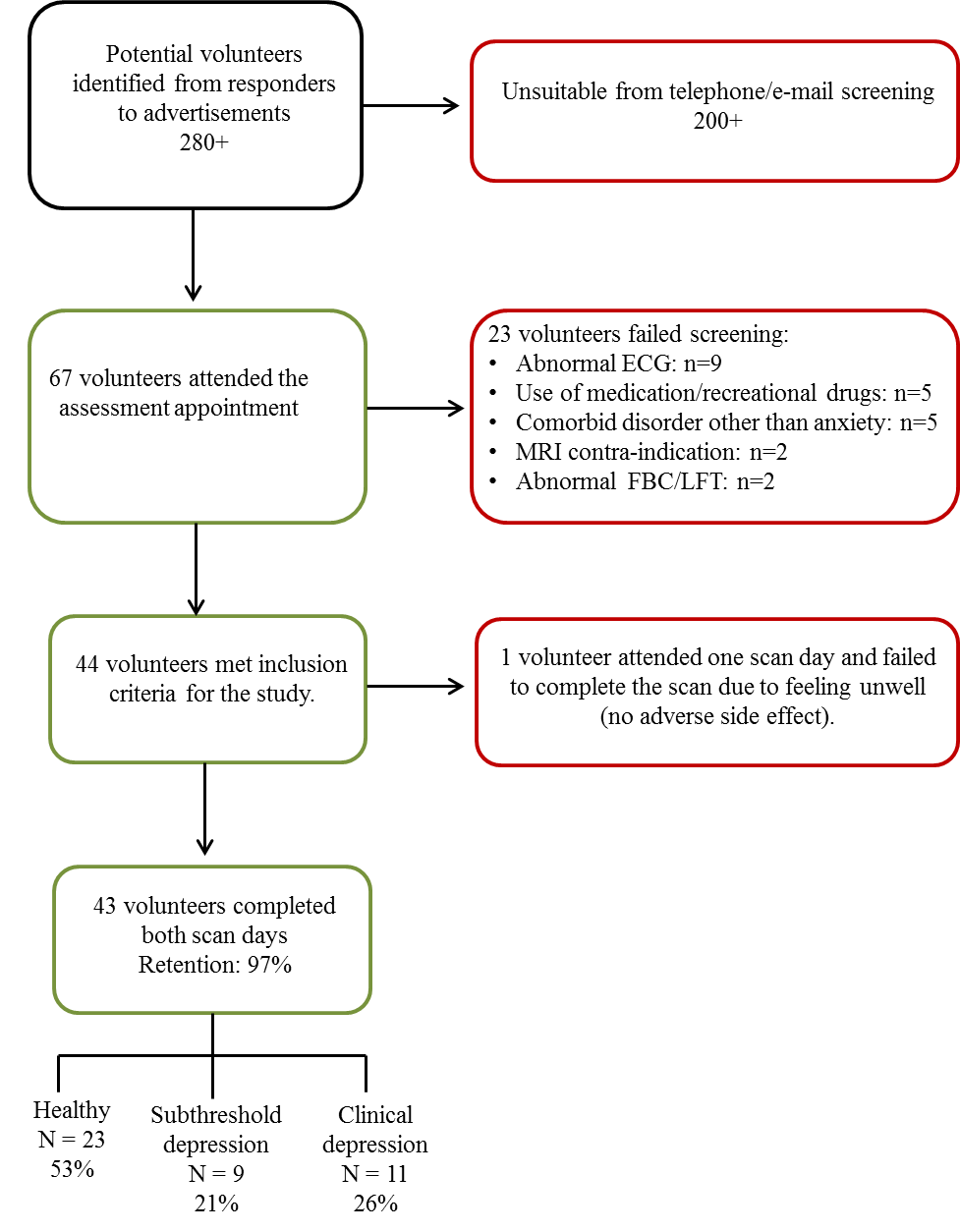


**Figure** **S1.** Flow chart illustrating the recruitment process. Of the 280+ potential volunteers who responded to advertisements, 67 were invited for further screening at an assessment appointment. Of these, 44 volunteers met inclusion criteria for the study and 43 of 44 participants completed the study, thereby giving a retention rate of 97%.


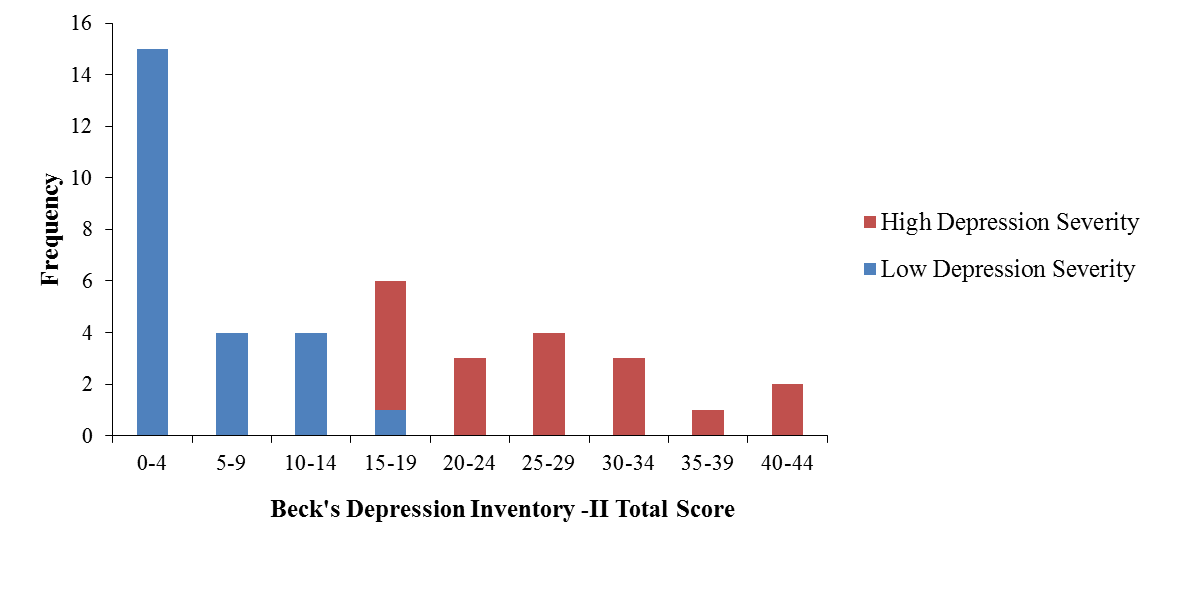


**
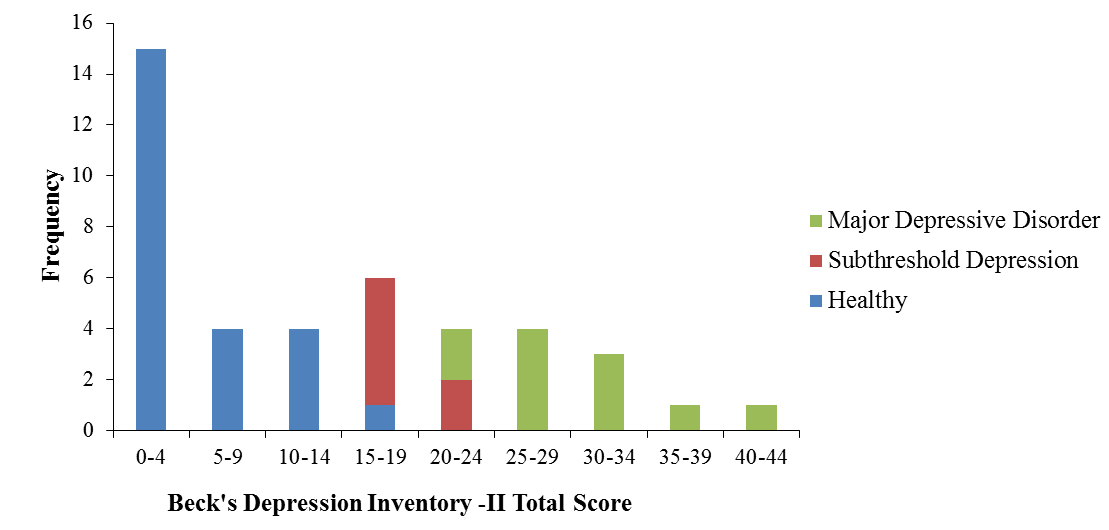
**

**Figure S2.** Histogram showing the frequency of Beck’s Depression Inventory (BDI-II) Total Scores in the sample organized by (A) Depression severity cut-off scores from the Beck’s Depression Inventory-II: individuals with *low depressive symptoms* (Total BDI-II score: 0-16 (normal-mild mood disturbance), n=24) versus *high depressive symptoms* (Total BDI-II score: 17-43 (borderline-severe depression), n=18); (B) Diagnosis as assessed by the Mini International Neuropsychiatric Interview version 6.0.0 (M.I.N.I): (*Healthy*: n=24, *Subthreshold Depression*: n=7, *Major Depressive Disorder*: n=11). The positive skew in the distribution of BDI-II scores in our sample is consistent with studies completed in the general population (Lasa *et al*, 2000).


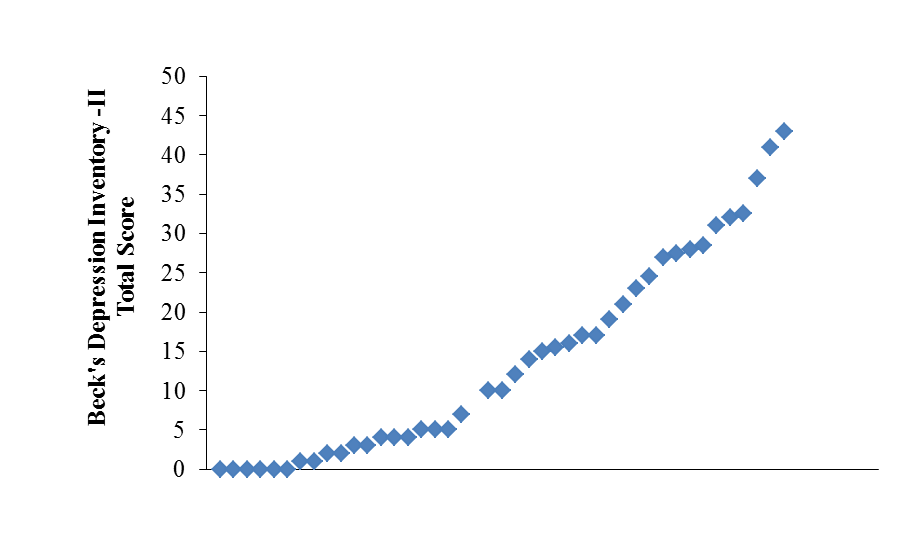


**Figure S3.** Scatterplot showing the distribution of Beck’s Depression Inventory (BDI-II) Total Scores in the sample.


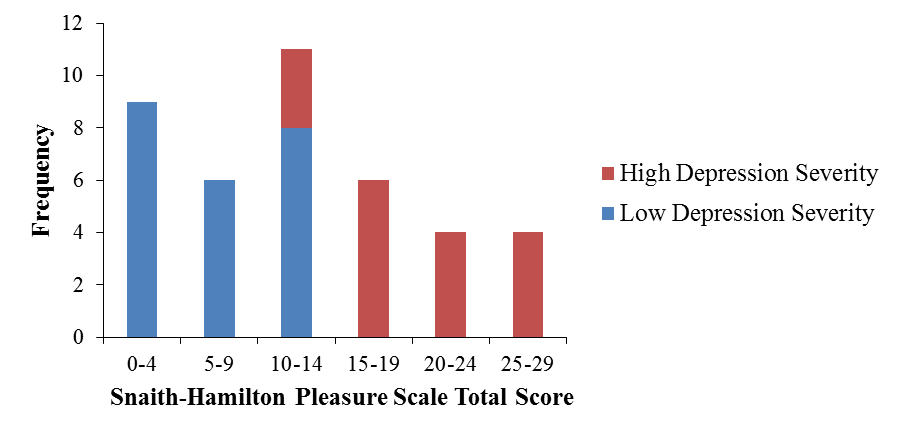


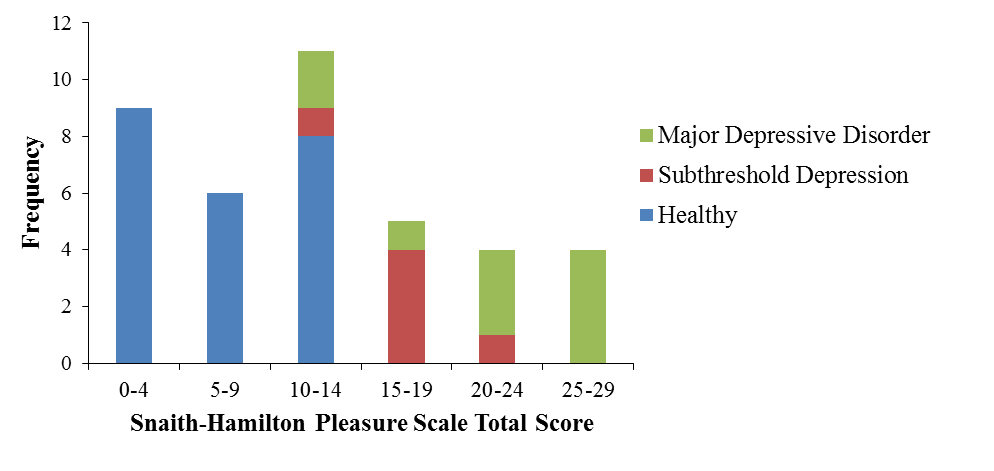


**Figure S4.** Histogram showing the frequency of Anhedonia Scores in the sample as measured by the Snaith-Hamilton Pleasure Scale (SHAPS) organized by (A) Depression severity cut-off scores from the Beck’s Depression Inventory-II: individuals with *low depressive symptoms* (Total BDI-II score: 0-16 (normal-mild mood disturbance), n=24) versus *high depressive symptoms* (Total BDI-II score: 17-43 (borderline-severe depression), n=18); (B) Diagnosis as assessed by the Mini International Neuropsychiatric Interview version 6.0.0 (M.I.N.I): (*Healthy*: n=24, *Subthreshold Depression*: n=7, *Major Depressive Disorder*: n=11).

**Table S1**. Demographic and clinical characteristics of recruited participants according to depression severity cut-off scores from the Beck’s Depression Inventory-II: individuals with *low depressive symptoms* (Total BDI-II score: 0-16 (normal-mild mood disturbance), n=24) versus *high depressive symptoms* (Total BDI-II score: 17-43 (borderline-severe depression), n=18). This table shows the convergence between BDI-II scores and depression diagnoses as assessed by the Mini International Neuropsychiatric Interview version 6.0.0 (M.I.N.I) (Sheehan *et al*, 1998).

| **Characteristic** | **High Depression Symptoms** ^a^  **(N=18)** | | **Low Depression Symptoms** ^b^  **(N=24)** | | **Test** | **p-value** |
| --- | --- | --- | --- | --- | --- | --- |
|  | Mean | SD | Mean | SD | T-test (dof) |  |
| Age (years) | 21.33 | 2.43 | 22.32 | 1.65 | 1.70(41) | .096 |
| Beck Depression Inventory – II | 26.44 | 8.79 | 4.86 | 5.01 | -8.58(39) | <.001 |
|  |  |  |  |  |  |  |
| Snaith-Hamilton Pleasure Scale | 19.12 | 6.01 | 7.21 | 5.99 | -6.69(39) | <.001 |
|  | N | % | N | % | χ^2^ |  |
| Female | 13 | 72.22 | 15 | 62.50 | .000 | .988 |
| Caucasian | 15 | 83.33 | 20 | 83.33 | .380 | .538 |
| Current subthreshold depression | 7 | 38.88 | 0 | 0.00 |  |  |
| Current MDD | 11 | 61.11 | 0 | 0.00 |  |  |
| Lifetime MDD | 15 | 83.33 | 2 | 8.33 |  |  |
| Lifetime MDD and Current subthreshold depression | 5 | 27.77 | 0 | 0.00 |  |  |
| Lifetime MDD and Current MDD | 10 | 55.55 | 0 | 0.00 |  |  |
| Current comorbid anxiety disorders | 10 | 55.55 | 0 | 0.00 |  |  |
| Individuals with  ^a^ *High Depression Severity* (Total BDI-II score: 17-43 (borderline- severe depression), n=18) and ^b^ *Low Depression Severity* (Total BDI-II score: 0-16 (normal-mild mood disturbance), n=24) according to depression severity cut-off scores for the BDI-II (Beck *et al*, 1996; Krefetz *et al*, 2002; Kumar *et al*, 2002).  All participants were right-handed, per inclusion criteria. MDD: Major Depressive Disorder.  Subthreshold and current and lifetime MDD and Anxiety Disorders diagnosed by the Mini International Neuropsychiatric Interview version 6.0.0 (M.I.N.I) (Sheehan *et al*, 1998). See Supplementary Methods for full details of diagnostic criteria. | | | | | | |

Pharmacological intervention: Lurasidone is a benzisothiazol derivative and has been classified as an atypical antipsychotic (AAP) (Greenberg and Citrome, 2017; Ishibashi *et al*, 2010)). The dose used for this study is the minimal effective dose for bipolar depression (20 mg) (Cates *et al*, 2013; Loebel *et al*, 2014) Time of administration three hours before scanning is intended to achieve a drug level comparable across groups and that is satisfactory for the scan acquisition (lurasidone is absorbed and reaches peak serum concentrations in 1-3 hours. This study is done in collaboration with the National Pharmacy at the Maudsley Hospital. They provide us with the lurasidone 20mg tablets and create the placebo tablets with identical appearance. Single-dose administration combined with imaging: This approach has been used successfully before (Handley *et al*, 2013; Ishibashi *et al*, 2010; Rock *et al*, 2013) and avoids sedation over long periods.

**Figure S5.** Lurasidone’s receptor binding profile.

**Interesting property of lurasidone:**

- Potential anxiolytic and antidepressant activity.

(Horisawa et al., 2013, Cates et al., 2013, Ishibashi et al., 2010)

**Characteristic of AAPs:**

- Potential antipsychotic function (Ishibashi et al., 2010)

**Interesting properties of lurasidone:**

- Potentially responsible for limited side effects eg. weight gain.

(Ishibashi et al., 2010, Sanford and Dhillon, 2015)

- Potential anxiolytic and antidepressant activity.

(Alamo et al., 2014, Ishibashi et al., 2010)

**The Monetary Incentive Delay Task**

The Monetary Incentive Delay (MID) task used in the present study was an adaptation of the task from Knutson et al., (2001) (Knutson *et al*, 2001). The task is a cued reaction time test for which symbolic cues signal the possibility of winning or losing money. Performance variation is minimized by emphasizing the need for response speed on all trials and by a tracking procedure that maintains performance at approximately 66% (Knutson *et al*, 2001). Specifically, task difficulty (i.e. the duration of the target appearance) was adapted to the performance of the subject, such that performance would be successful (reaction within the interval that the target was shown on screen) on approximately 66% of trials. The task used in the current study included these features. Each trial consisted of anticipation, response, and feedback. During the anticipation phase, participants were presented with one of three cue shapes (cue, 250 ms) denoting the type of reward that could be attained by a correct response (Figure S6). After a variable anticipation interval (3,800–4,150 ms), the target (white square) appeared on the centre of the screen. Participants were instructed to respond to the target as quickly as possible by pressing a button with their right index finger.

On the first trial, the target was displayed for 400 ms, and display durations for subsequent targets varied from trial to trial in 10-ms steps (150–500 ms), depending on subject performance. Responses made during the anticipation period and responses less than 100 ms were considered as "premature presses". The maximum response duration was 700 ms and reaction times greater than 700 ms were not recorded. A variable delay (blank screen) appeared after the target presentation and before feedback.

Following the response, feedback was given (1,450 ms), indicating how much money was won or lost during the trial and the total money earned during the task. As shown in Figure S6, the striped circle symbol denoted a ‘win trial’. Participants received £1 for a successful response (‘win’), and £0 for an unsuccessful response (‘missed win’). The striped square symbol denoted a ‘loss trial’. Following a successful response, participants avoided losing £1 (‘avoided loss’), and lost £1 if their reaction was outside the target response window (‘loss’). The triangle symbol denoted a ‘neutral trial’ in which outcomes were not contingent on making a response within the target response window. Participants did not gain or lose money and their earnings remained £0, regardless of a successful or unsuccessful response. The screen went blank after the feedback for a variable delay (2950 ms-3300 ms) to take each trial to 9500 ms in length.

In addition to the ‘Neutral’, ‘Win’ and ‘Loss’ trials, in which a motor response was required (i.e. ‘active trials’), the task included passive trials in which no motor response was required. An ‘X’ cue was presented on screen (250 ms), followed by a blank screen (4000 ms) (trial length: 4250 ms). The rationale for including a passive condition is to pilot an alternative baseline condition to the ‘neutral cue’. Participants are presented with a symbol which requires no motor response, and it may be possible to separate out the neurocognitive processes underlying ‘the preparation of a motor response’ from ‘the anticipation of a reward’.

‘Neutral’, ‘Win’, ‘Loss’ and ‘Passive’ conditions were randomized throughout the task (13 trials each, summing up to 39 9500 ms ‘active trials’ and 13 4250 ms ‘passive trials’ in total). Task running time was 7 minutes 10 seconds. Participants completed two runs of the task at the placebo visit and the Lurasidone visit to ensure that there were a sufficient number of trials (26 trials) per condition for analysis and to reduce boredom effects. There was a short pause between the two runs of the task.

At the assessment appointment (first visit), participants learnt the meanings of the four symbol cues and completed a practice session of the MID task in a mock scanner for approximately 7 minutes. Participants were asked to recall the meaning of each symbol prior to each scanning session and the need for response speed on all trials was emphasized by the experimenter. Participants received their winnings in cash at the end of the task.


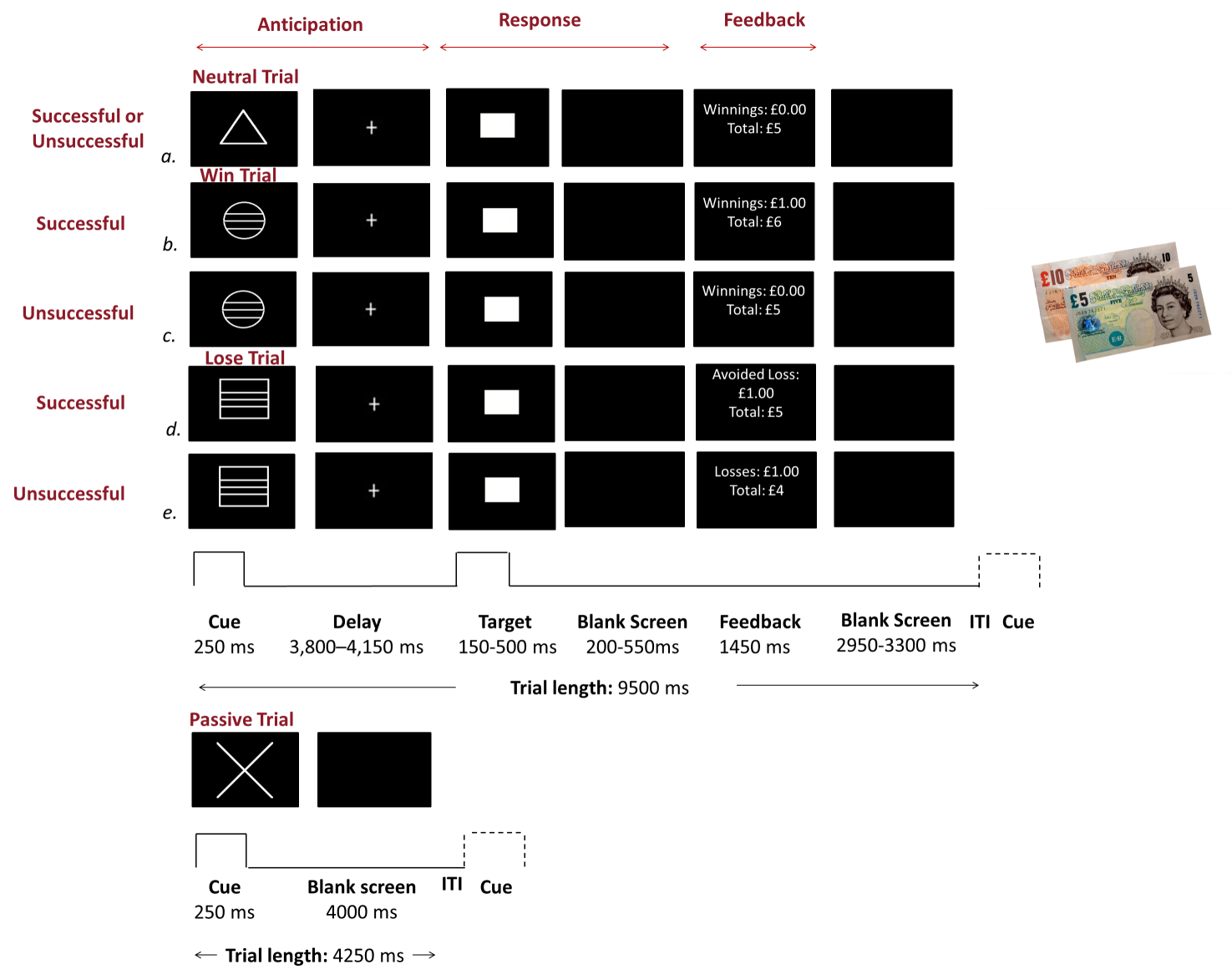


**Figure S6.** Monetary Incentive Delay Task.

**MRI acquisition**

Magnetic resonance data were acquired on a 3Tesla MR scanner (GE 750 Signa Excite) with a 12-channel head coil.

*Arterial Spin Labelling*

In Arterial Spin Labelling (ASL), the MR signal of water in arterial blood is selectively inverted using an external radiofrequency pulse applied in the region of the carotid arteries, in order to achieve total inversion of the arterial input to the brain. In the pulse sequence used in this study, inversion was achieved using the method known as “pseudo-continuous ASL”, introduced by Alsop (Dai *et al*, 2008). During a delay time known as “post-labelling delay”, this labelled arterial magnetisation enters the brain volume and disperses through the arterial network and eventually reaches the tissue capillaries. The delay time is made sufficiently long in order to make sure that most of the labelled spins reside only in the capillary domain. At this time, a whole brain image is acquired as fast as possible. Two, whole brain images are acquired (one with arterial blood labelling as described above and one without). If the labelled image is subtracted from the non-labelled one, the voxel-wise difference values are proportional to the volume of arterial blood that flows into each volume element during the post-labelling delay. Using a reference image and an appropriate mathematical model, the voxel-wise difference values are converted to a whole brain map of Cerebral Blood Flow (CBF) in traditional physiological units of ml blood/100gm tissue/min (Dai *et al*, 2011). The continuous pair-wise subtraction of labelled and non-labelled images exhibits minimal sensitivity to low frequency signal drift.

Labelling of arterial blood was achieved with a 1.5 second long RF pulse consisting of 1500 Hanning shaped RF pulses of 500us duration, applied in the presence of a net field gradient in the direction of flow (the ‘z’axis). A post-labelling delay of 1.5s was used, which included background suppression RF pulses to minimise the static tissue signal. Images were acquired using a 3D FSE, multi-shot ‘stack of spirals’ protocol, employing 8 spiral arms for each inter-leave and three ‘control-labelled’ averages. The images had 54 slice locations (3mm thickness, no inter-slice gap) and an in-plane resolution of 1mm after transformation to a rectangular grid (TE/TR = 11.088/4901ms, flip angle (FA) = 111°). A proton density image volume with the same parameters was acquired in order to use as a reference to compute the CBF in physiological units.

*Structural images*

High-resolution T1-weighted sagittal ADNI GO images were acquired [TR = 7.3ms; TE = minimum full; FOV = 270mm; matrix = 256x256mm; 1.2mm slice thickness; 200 slices]. T2 images were acquired using FRFSE-XL forced recovery fast spin echo [TR = 4380ms; TE = 60.0ms; FOV = 240mm; matrix frequency 320, zipped to 512 phase 256; slice thickness 1.2mm with 0mm spacing; 72 slices]. The T1 and T2 images were used in pre-processing.

*Functional MRI for MID Task*

For each subject and at each visit, 216 whole-brain volumes (41 slices with continuous descending acquisition aligned to the AC–PC plane) were acquired using an EPI-GE sequence [Repetition Time (TR) = 2000ms, Echo Time (TE) = 30ms, flip angle = 75º, slice thickness = 3.0 mm, interslice gap = 0.3 mm and matrix size = 64x64mm]. Duration of the sequence was 7minutes 20 seconds.

**fMRI Data Analysis**

ASL pre-processing:

First, CBF maps were co-registered with a T1-weighted anatomical image after coarse alignment of the origin of both images. Unified segmentation of the T1-weighted image normalised this image to the MNI space and was used to produce a ‘brain-only’ binary mask which was multiplied by the co-registered rCBF map to produce an image free of extra-cerebral artefacts. The spatial transformation matrix was applied to the clean CBF images and then smoothed using an 8x8x8mm kernel.

**Movement Analyses**

Movement analyses showed that the maximum number of volumes lost for spikes greater than 1mm was 9% (19 of 216 volumes) and all sessions were included. A repeated measures ANCOVA with two within-subject factors: *Movement* (number of spikes or total movement) and *Medication* (placebo, lurasidone), one between subject factor: *Medication Order*, and *Depression Severity* (total BDI score) as the covariate of interest, showed that there were no significant *Medication*-*Movement* or *Depression Severity*-by-*Movement*, or *Medication*-by-*Depression Severity*-by-*Movement* interactions (all *p* values >.050). There were no significant interactions with *Medication Order* (all *p* values >.050).

**Whole-brain analysis**

Whole-brain analyses modelled effects of lurasidone and depression status beyond the fronto-striatal network targeted in the ROI analyses. Within-subject contrasts were calculated at the first-level for each participant (placebo>lurasidone) and carried forward for a whole brain independent samples t-test at second level with *Medication Order* (placebo-lurasidone, lurasidone-placebo) as the between subject factor and *Depression Severity* as the covariate of interest. The independent samples t-test was conducted separately for the responses to: (i) anticipation neutral>baseline (ii) anticipation win>baseline (ii) anticipation loss>baseline (iv) feedback win>missed win and (v) feedback loss>avoided loss. In addition, whole brain analyses of the entire sample treated as a single group (n=84) were conducted for each condition relative to baseline on placebo (i.e., Reward Cue, Penalty Cue, Reward Outcome and Penalty Outcome). This analysis was completed to test whether the task elicited the expected pattern of activation (Knutson *et al*, 2001).

Results were reviewed with an initial voxel threshold of *p*<.001 and cluster corrected to *p*<.05 by being required to exceed an extent of 116 continuous voxels, as determined by AFNI’s 3dClustSim (Cox *et al*, 2017; Eklund *et al*, 2016). For all comparisons, brain locations were reported as x, y, and z coordinates in Montreal Neurologic Institute (MNI) space and Wake Forest University (WFU) PickAtlas was used to identify brain regions.

**Supplementary Results**

**Behavioural results**

There were no significant interactions between *Total Winnings* and *Depression Severity* (*F*=.715, df=1, 40, *p*=.403), *Total Winnings* and *Medication* (*F*=.038, df=1, 40, *p*=.847), or *Total Winnings*-by-*Depression Severity*-by-*Medication* (*F*=.038, df=1, 40, *p*=.480).

Analysis of mean RT revealed a *Cue Type*-by-*Mean RT* interaction (*F*=55.39, df=2, 74, *p*>.001), with no interactions with *Medication* or three-way interactions (all *p* values > .050).The effect of cue type was driven by longer reaction time to neutral cues relative to either reward cues (*p* < 0.001) or penalty cues (*p* < 0.001). This reflects motivated responding on reward and penalty trials versus neutral trials across the entire sample. The same pattern remained when separating the sample into groups using medication type and BDI median split (Low Depression Severity: BDI scores 0-16; High Depression severity: BDI scores >17).

Analysis of accuracy showed a significant *Cue Type*-by-*Accuracy* interaction (*F*=55.39, df=2, 80, *p*>.001), with no interactions with *Medication* or three-way interactions (all *p* values > .050). Post-hoc tests showed that the effect of cue type was due to lower accuracy to neutral cues relative to either reward cues (*p* < 0.001) or penalty cues (*p* < 0.001). The groups also did not differ in the percentage of reward trials ending in gains or the percentage of loss trials ending in penalties (i.e. *Outcome Frequency*). Specifically, a mixed-effects ANOVA with *Outcome* *Type* (Win, No-Win, Loss, No-Loss, No Change) and *Medication* (placebo, lurasidone) as within-subject variables, *Medication Order* as a between-subject variable, and *Depression Severity* as the covariate of interest, revealed only a main effect of *Outcome Type* (*F*= 39.06, df= 2 , 80, *p* < 0.001). Post-hoc contrasts showed that this main effect was due to a higher frequency of Win relative to Missed Win outcomes following a reward cue (*p* < 0.001), and a higher frequency for Avoided Loss relative to Loss following the penalty cue (*p* < 0.001). These results are consistent with the task tracker which intends to ensure approximately 66% successful trials – Win or Avoided Loss – for all participants. The analyses of the reaction time and accuracy (outcome frequency), suggest that the BOLD fMRI findings were not confounded by group differences in task difficulty.

We also examined the effect of *Medication*, *Medication Order* and *Depression Severity* on the change in *Sedation* ratings (Total VAS scores) and *State-anxiety* ratings (total STAI score) from pre-drug administration (Measure 1) to peak-of-drug (Measure 2). Interactions between *Medication* and *State-anxiety* (*F*=.143, df=1, 37, *p*=.708) and interactions between *Medication* and *Sedation ratings* (*F*=.199, df=1, 40, *p*=.658) were non-significant. There were no significant interactions with *Medication Order* (all *p* values > 0.05). Three way interactions between *Medication, Depression Severity* and *Sedation* (*F*=1.34, df=1, 40, *p*=.253), and, *Medication, Depression Severity* and *State-anxiety* (*F*=.038, df=1, 40, *p*=.480) were also non-significant. These results suggest that lurasidone did not lead to a significant change in sedation or state anxiety relative to placebo over a period of three hours, and that this was consistent across the continuum of depression severity.

**Reward Processing (Blood-Oxygen-Level Dependent signal) Results**

Three participants were excluded from this analysis for the following reasons (i) one (low depression severity) participant did not adhere to the requirements of the task (omitted responses to neutral cues on one run of the MID task at the placebo visit); (ii) one (low depression severity) participant had ACC mean activation values that were greater than three standard deviations from the group mean for the *Penalty Outcome* contrast, and (iii) one (high depression severity) participant had ACC and NAcc mean activation values that were greater than three standard deviations from the group mean for the *Reward Outcome* contrast.

*Response to Outcomes*

The *Medication*-by-*Depression Severity*-by-*Penalty Outcome* interaction in the OFC (*F*=4.94, df=1, 37, *p*=.032) showed a similar pattern of results to the ACC findings, but fell short of significance after Bonferroni correction for seven multiple ROI comparisons (significance level at *p*=.007). The *Medication*-by-*Depression Severity*-by-*Reward Outcome* interaction displayed a pattern of signal normalisation in the Nucleus Accumbens, however, it fell short of significance (*F*=4.87 df=1, 38 *p*=.033) following Bonferroni correction.

These analyses were repeated using continuous anhedonia scores from the SHAPS questionnaire; however, all *Medication*-by-*Depression severity*-by-*Outcome* *Type* interactions were non-significant.

*Inclusion of outliers (two subjects)*

All analyses for responses to outcomes were repeated including the two participants with outlier values and all results remained the same. First, including outlier values in the repeated measures ANCOVA (n=42), did not change the pattern or significance of the *Medication*-by-*Depression severity*-by-*Outcome* *Type* interaction in the ACC, OFC and insula, according to the Bonferroni-corrected significance threshold *(p*<.007): ACC (F=12.99, df=1,39, *p*=.001), orbitofrontal cortex (OFC) (*F*=4.51, df=1, 39, *p*=.040) and Insula *(F*=4.75, df=1, 39, *p*=.035). Second, the pattern or significance of the *Medication*-by-*Depression Severity*-by-*Penalty Outcome* interaction in the ACC and OFC remained the same (without outlier: ACC (*F*=11.98, df=1, 38, *p*=.001); OFC (*F*=4.94, df=1, 37, *p*=.032); with outlier: ACC (*F*=13.69, df=1, 39, *p*=.001) OFC (*F*=6.83, df=1, 39, *p*=.013)). Third, the *Medication*-by-*Depression Severity*-by-*Reward Outcome* interaction did not change (without outlier: the NAcc (*F*=4.87 df=1, 38 *p*=.033) and ACC (*F*=5.92 df=1, 37, *p*=.020); with outlier: NAcc (*F*=5.65, df=1, 39, *p*=.022) and ACC (*F*=7.32, df=1, 39, *p*=.010).

*Anxiety severity analyses*

A repeated measures ANCOVA was completed for the anticipation phase (*Cue Type* (Reward, Penalty, Neutral)) and outcome phase (*Outcome Type* (*Reward Outcome* versus *Penalty Outcome*)) of the task with *Medication* (placebo or lurasidone) as the within-subject factor, *Medication Order* (placebo-lurasidone, lurasidone-placebo) as the between-subject factor; and *Anxiety Severity* (total Anxiety score from the HADS) as the covariate of interest. There were no significant associations between brain activity and anxiety and no significant effects with anxiety severity after Bonferroni correction (anticipation: ACC (*F*=0.01, df=2,74, *p*=.995), outcome: ACC (*F*=5.92, df=1,37, *p*=.020). Therefore, anxiety severity was not included as a covariate in the ANCOVA model with depression severity (Miller and Chapman, 2001).

*Whole-brain analysis*

The independent samples t-test demonstrated that there were no significant clusters or voxels for the interaction of *Medication, Medication Order* and all contrasts of interest. There were no significant associations between *Medication* and *Depression Severity* for the contrasts: (i) anticipation neutral>baseline (ii) anticipation win>baseline (iii) anticipation loss>baseline and (iv) feedback win>missed win. Using an un-corrected threshold, a cluster of 64 voxels in the ACC (x=2,y=34,z=6, t=4.34, *p*=.046) showed a positive correlation between depression severity and the contrast ‘feedback loss>avoided loss’ (placebo > lurasidone).

**Table S2.** Performance data of the Monetary Incentive Delay (MID) task, split by Medication

|  | Medication, Mean (SD) | | Repeated Measures ANCOVA(*Medication*-by-*Depression Severity*) | |
| --- | --- | --- | --- | --- |
|  | **Placebo** ^a^ | **Lurasidone** ^b^ | **F value** | **P value** |
| **Total Winnings, (£)** | 17.98(4.42) | 17.70(4.51) | 0.05 | .463 |
| **Success, %** |  |  |  |  |
| Overall | 59.24(6.09) | 59.15(5.86) | 0.11 | .740 |
| Neutral trials | 47.05(8.91) | 47.85(9.01) | 0.58 | .452 |
| Win Trials | 68.25(9.27) | 67.71(9.94) | 0.45 | .506 |
| Loss trials | 62.43(10.08) | 61.90(11.07) | 0.24 | .624 |
| **Reaction time, ms** |  |  |  |  |
| Neutral Outcome | 269.70(26.78) | 275.70(27.79) | 0.17 | .681 |
| Missed Neutral Outcome | 319.40(50.43) | 335.70(59.13) | 0.20 | .659 |
| Reward/Win Outcome | 253.00(21.75) | 259.80(25.38) | 0.31 | .580 |
| Missed Win Outcome | 273.10(52.23) | 292.60(51.67) | 3.98 | .054 |
| Penalty/Loss Outcome | 269.80(46.12) | 293.60(55.59) | 0.00 | .960 |
| Avoided Loss Outcome | 249.60(23.19) | 253.90(23.22) | 0.05 | .824 |
| **Change** ^c^ **in Sedation** | -26.19(141.42) | -85.07(151.40) | 1.18 | .284 |
| **Change** ^c^ **in State-Anxiety** | 0.52(4.88) | -0.95(3.78) | 3.64 | .064 |
| a Individuals with Low Depression Severity (BDI scores <16) (n=24) and High Depression Severity (BDI scores ≥17) (n=18) on Placebo ( total n=42).  b Individuals with Low Depression Severity (BDI scores <16) (n=24) and High Depression Severity (BDI scores ≥17) (n=18) on Lurasidone (n=42).  c Change score from pre-drug administration (Measure 1) to peak-of-drug (Measure 2), approximately three hours after drug administration.  A repeated measures ANCOVA with Medication (placebo or lurasidone) as the within-subject variable, Medication Order (placebo-lurasidone, lurasidone-placebo) as the between subject variable and *Depression Severity* (total BDI score) as the covariate interest was completed for (i) *Total Winnings*, (ii) *Accuracy* and (iii) *Mean Reaction Time (RT)* to the target on win, neutral and loss trials. | | | | |

**Figure S7.** Anatomical masks: Location of anatomically defined masks for the Caudate (turquoise), Nucleus Accumbens (NAcc) (yellow), and Putamen (maroon), Orbitofrontal Cortex (OFC) (red), Insula (Green), Amygdala (Indigo), Anterior Cingulate Cortex (ACC) (Blue). The Caudate, putamen, Insula and ACC ROIs were formed from the AAL atlas in SPM. The OFC ROI was formed using Brodmann Area 13 and the NAcc from the IBASPM71 atlas in SPM12.


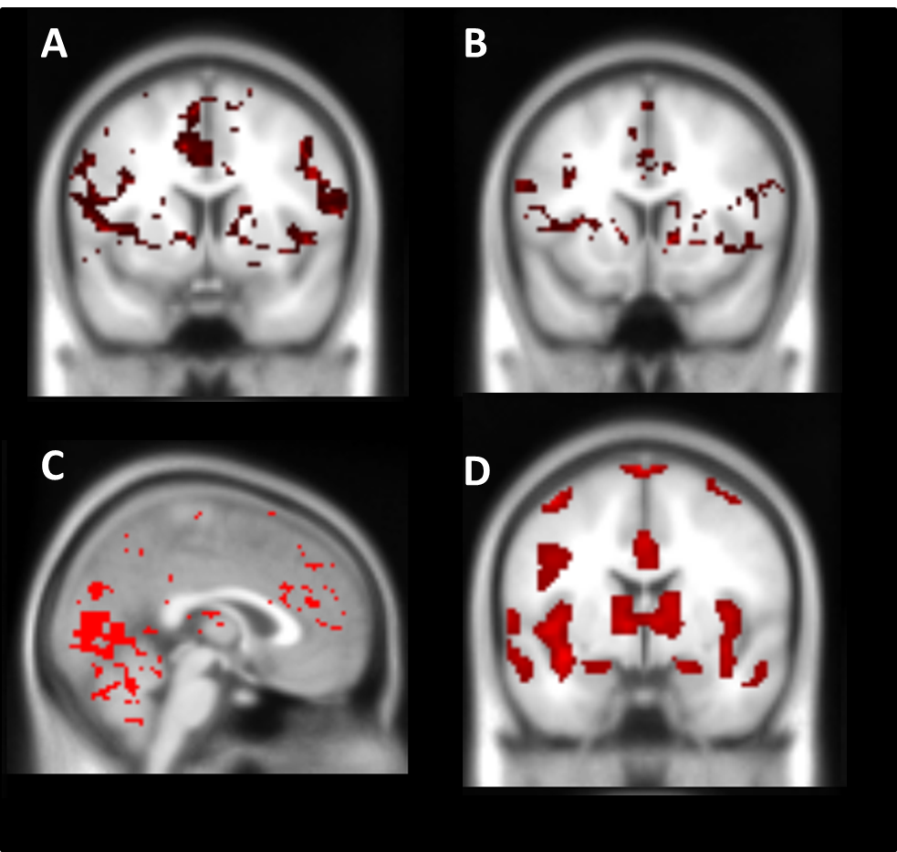


**Figure S8.** Whole Brain Responses to Reward and Penalty cues and outcomes across the entire sample.

Whole-brain analysis: Whole-brain analyses across the entire sample (n= 84) in response to anticipation and receipt of monetary rewards and penalties revealed the expected pattern of activation. Specifically, in response to (A) reward anticipation and (B) penalty anticipation, robust activation was observed across striatum, insula, hippocampus and motor preparation regions. In response to receipt of monetary rewards (C) and penalties (D) participants exhibited activity in the ACC, cerebellum and visual cortex. All whole-brain analyses were thresholded and cluster corrected using the same thresholds (cluster corrected to *p*<.050 by being required to exceed an extent of 116 continuous voxels, as determined by AFNI’s 3dClustSim (Cox *et al*, 2017; Eklund *et al*, 2016).

**References**

**Alamo C, Lopez-Munoz F, Garcia-Garcia P** (2014). The effectiveness of lurasidone as an adjunct to lithium or divalproex in the treatment of bipolar disorder. *Expert Review of Neurotherapeutics* **14**, 593-605.

**Beck AT, Steer RA, Ball R, Ranieri WF** (1996). Comparison of Beck Depression Inventories-IA and -II in psychiatric outpatients. *Journal of Personality Assessment* **67**, 588-597.

**Cates LN, Roberts AJ, Huitron-Resendiz S, Hedlund PB** (2013). Effects of lurasidone in behavioral models of depression. Role of the 5-HT7 receptor subtype. *Neuropharmacology* **70**, 211-217.

**Cox RW, Chen G, Glen DR, Reynolds RC, Taylor PA** (2017). FMRI Clustering in AFNI: False-Positive Rates Redux. *Brain connectivity* **7,** 152-171.

**Dai W, Garcia D, de Bazelaire C, Alsop DC** (2008). Continuous Flow-Driven Inversion for Arterial Spin Labeling Using Pulsed Radio Frequency and Gradient Fields. *Magnetic Resonance in Medicine* **60**, 1488-1497.

**Dai W, Robson PM, Shankaranarayanan A, Alsop DC** (2011). Sensitivity Calibration With a Uniform Magnetization Image to Improve Arterial Spin Labeling Perfusion Quantification. *Magnetic Resonance in Medicine* **66**, 1590-1600.

**Eklund A, Nichols TE, Knutsson H** (2016). Cluster failure: Why fMRI inferences for spatial extent have inflated false-positive rates. *Proceedings of the National Academy of Sciences of the United States of America* **113**, 7900-7905.

**Greenberg WM, Citrome L** (2017). Pharmacokinetics and pharmacodynamics of lurasidone hydrochloride, a second-generation antipsychotic: a systematic review of the published literature. *Clinical Pharmacokinetics* **56**, 493-503.

**Handley R, Zelaya FO, Reinders AATS, Marques TR, Mehta MA, O'Gorman R** (2013). Acute effects of single-dose aripiprazole and haloperidol on resting cerebral blood flow (rCBF) in the human brain. *Human Brain Mapping* **34**, 272-282.

**Horisawa T, Ishiyama T, Ono M, Ishibashi T, Taiji M** (2013). Binding of lurasidone, a novel antipsychotic, to rat 5-HT7 receptor: Analysis by H-3 SB-269970 autoradiography. *Progress in Neuro-Psychopharmacology & Biological Psychiatry* **40**, 132-137.

**Ishibashi T, Horisawa T, Tokuda K, Ishiyama T, Ogasa M, Tagashira R** (2010). Pharmacological Profile of Lurasidone, a Novel Antipsychotic Agent with Potent 5-Hydroxytryptamine 7 (5-HT(7)) and 5-HT(1A) Receptor Activity. *J Pharmacol Exp Ther* **334**, 171-181.

**Knutson B, Adams CM, Fong GW, Hommer D** (2001). Anticipation of increasing monetary reward selectively recruits nucleus accumbens. *Journal of Neuroscience* **21**, 1-5.

**Krefetz DG, Steer RA, Gulab NA, Beck AT** (2002). Convergent validity of the Beck Depression Inventory-II with the Reynolds Adolescent Depression Scale in psychiatric inpatients. *Journal of Personality Assessment* **78**, 451-460.

**Kumar G, Steer RA, Teitelman KB, Villacis L** (2002). Effectiveness of Beck Depression Inventory-II subscales in screening for major depressive disorders in adolescent psychiatric inpatients. *Assessment* **9**, 164-170.

**Lasa L, Ayuso-Mateos JL, Vazquez-Barquero JL, Diez-Manrique FJ, Dowrick CF** (2000). The use of the Beck Depression Inventory to screen for depression in the general population: a preliminary analysis. *Journal of Affective Disorders* **57**, 261-265.

**Lewinsohn PM, Solomon A, Seeley JR, Zeiss** A (2000). Clinical implications of "subthreshold" depressive symptoms. *Journal of Abnormal Psychology* **109**, 345-351.

**Loebel A, Cucchiaro J, Silva R, Kroger H, Hsu J, Sarma K** (2014). Lurasidone monotherapy in the treatment of bipolar I depression: a randomized, double-blind, placebo-controlled study. *The American Journal of Psychiatry* **171**, 160-168.

**Rock PL, Harmer CJ, McTavish SFB, Goodwin GM, Rogers RD** (2013). Short-Term Quetiapine Treatment Alters the Use of Reinforcement Signals during Risky Decision-Making and Promotes the Choice of Negative Expected Values in Healthy Adult Males. *Journal of Neuroscience* **33**, 15588-15595.

**Sanford M, Dhillon S** (2015). Lurasidone: A Review of Its Use in Adult Patients with Bipolar I Depression. *CNS Drugs* **29**, 253-263.

**Sheehan DV, Lecrubier Y, Sheehan KH, Amorim P, Janavs J, Weiller E** (1998). The Mini-International Neuropsychiatric Interview (MINI): The development and validation of a structured diagnostic psychiatric interview for DSM-IV and ICD-10. *Journal of Clinical Psychiatry* **59**, 22-33.
